# Neuroprotective Effects of Early TLR4 Blockade with Compound C34 in Temporal Lobe Epilepsy: Alleviation of Neuroinflammation and Apoptosis

**DOI:** 10.5812/ijpr-159165

**Published:** 2025-03-08

**Authors:** Roya Varmazyar, Nima Naderi, Hanieh Javid, Rasoul Ghasemi, Hamid Gholami Pourbadie

**Affiliations:** 1 Neuroscience Research Center, Institute of Neuroscience and Cognition, Shahid Beheshti University of Medical Sciences, Tehran, Iran; 2 Department of Pharmacology and Toxicology, Faculty of Pharmacy, Shahid Beheshti University of Medical Sciences, Tehran, Iran; 3 Department of Physiology, Neurophysiology Research Center, School of Medicine, Shahid Beheshti University of Medical Sciences, Tehran, Iran; 4 Department of Physiology and Pharmacology, Pasteur Institute of Iran, Tehran, Iran

**Keywords:** Apoptosis, C34, NF-κB1, Pilocarpine, Status Epilepticus, TLR4, TNF-a

## Abstract

**Background:**

Temporal lobe epilepsy (TLE) is a chronic neurological disorder characterized by hippocampal necrosis and apoptosis. Neuroinflammation plays a critical role in the pathophysiology of TLE, with toll-like receptor 4 (TLR4) serving as a key mediator. Activation of TLR4 leads to the release of pro-inflammatory cytokines, such as IL-6 and TNF-α, which contribute to neuronal injury and apoptosis. The TLR4 signaling pathway promotes neuroinflammation through nuclear factor kappa-B (NF-κB) activation, further exacerbating neuronal damage over time. Therefore, timely inhibition of TLR4 may help mitigate neuroinflammation and alleviate epilepsy symptoms.

**Objectives:**

This study aimed to determine whether early inhibition of TLR4 can regulate seizures and apoptosis by targeting the NF-κB1 signaling pathway.

**Methods:**

The TLR4 inhibitor C34 was administered intraventricularly to two experimental groups. The first group received the injection immediately after pilocarpine-induced seizures, while the second group was treated 24 hours post-pilocarpine injection. The expression levels of NF-κB1, TNF-α, and caspase-3 were analyzed using western blotting. Neuronal death in the hippocampus was assessed using hematoxylin and eosin (H&E) staining.

**Results:**

The results demonstrated that early inhibition of TLR4 by C34, administered immediately after seizure induction, significantly reduced NF-κB1, TNF-α, and caspase-3 expression levels compared to the group that received C34, 24 hours later. Additionally, early treatment with C34 significantly prevented pilocarpine-induced neuronal death in the hippocampus compared to the late treatment group.

**Conclusions:**

These findings highlight the importance of early intervention in reducing neuronal death and suppressing neuroinflammation in an epilepsy model. Inhibiting TLR4 immediately after seizure induction may serve as a potential therapeutic strategy to minimize inflammation-mediated neuronal damage in TLE. Further research is needed to explore the long-term effects of TLR4 inhibition in epilepsy treatment.

## 1. Background

Epilepsy is among the most prevalent neurological disorders, affecting over 70 million individuals globally. It is characterized by recurrent seizures due to spontaneous abnormal neuronal discharges in the brain (1). Temporal lobe epilepsy (TLE) is a subtype of epilepsy involving degeneration and reorganization of hippocampal networks (2). Approximately 30% to 50% of TLE patients do not respond to antiseizure medication therapy and are classified as having drug-resistant epilepsy(DRE) (3). The mechanisms underlying resistance to anti-seizure drugs remain incompletely understood. A significant factor in TLE drug resistance is neuronal cell loss and synaptic reorganization in the hippocampal region, known as hippocampal sclerosis (HS) (4). Neuroinflammation also contributes to the development of TLE (5). Evidence suggests that neuroinflammation plays a crucial role in the etiopathogenesis of epileptic seizures, as shown in both clinical trials and animal studies. Levels of pro-inflammatory cytokines, such as interleukin-1β (IL-1β), IL-2, IL-6, and tumor necrosis factor-α (TNF-α), increase significantly following epileptic seizures (6). Seizure-induced brain injuries alter its structure and function, leading to aberrant neurogenesis and heightened neuroinflammatory responses (7). The rise in pro-inflammatory mediators within the epileptic focus of the temporal lobe induces apoptosis and promotes further degeneration, potentially contributing to antiepileptic drug resistance in affected patients (8).

Recently, toll-like receptor 4 (TLR4) has garnered attention for its role in neuroinflammation. The TLR4-mediated inflammatory signaling pathway may be involved in DRE in rats, leading to elevated levels of TLR4, NF-κB, IL-1β, and TNF-α expression (9). A reduction in TLR4, NF-κB, and IL-1β expression correlates with decreased spontaneous chronic seizures in pilocarpine-induced epileptic rats (10). Inhibiting the IL-1R1/TLR4 pathway has been identified as a potential therapeutic target in animal models of acquired epilepsy (11). Current anti-seizure medications (ASMs) primarily provide symptomatic relief; while they can prevent seizure recurrence, they do not address the underlying causes of epilepsy or halt disease progression (5). Thus, research into effective preventive and curative measures for epilepsy remains crucial, as does the timing of treatment initiation. Immediate treatment after the first unprovoked seizure may reduce relapse risk but does not significantly affect the long-term prognosis of epilepsy (12).

## 2. Objectives

In this study, we demonstrate the importance of treatment timing in epilepsy. Our objective was to compare early treatment of the pilocarpine epilepsy model through TLR4 inhibition with late treatment by evaluating pro-inflammatory agents, apoptotic expression, neuronal death in hippocampal tissue, and mortality rates in rats under both early and late treatment conditions.

## 3. Methods

### 3.1. Animals

Adult male Wistar rats (n = 28, 200 ± 20 g) were utilized for the experiments. The rats were procured from the Pasture Institute of Iran and were group-housed in the Neuroscience Research Center animal facility. They were maintained on a 12-hour light/dark cycle at a temperature of 23 ± 2^°^C, with unrestricted access to standard rodent food and water, for two weeks prior to the commencement of the study to facilitate adaptation. All procedures involving animals were approved by the Iranian Council on Research Ethics and the Shahid Beheshti University of Medical Sciences Committee on Animal Care.

### 3.2. Experimental Design

**Figure 1.**
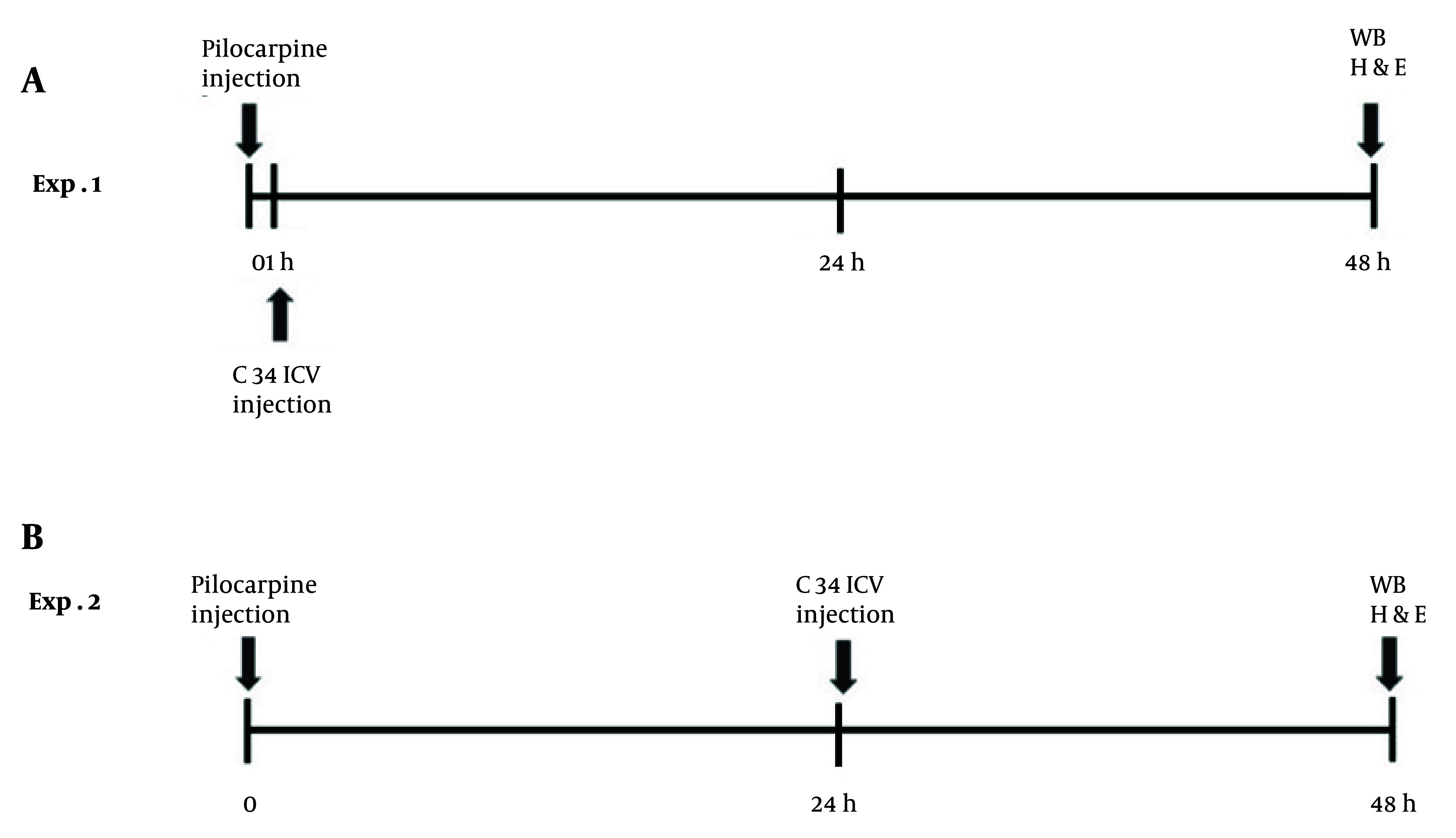
Diagram indicating the timeline of the experimental procedures in 2 groups of A, early treatment and B, late treatment

Rats were randomly assigned to four groups: (1) Control group: Rats received two saline injections, one intraperitoneal (IP; 100 µL/kg) and one intracerebroventricular[(ICV); injection volume 5 µL]; (2) epilepsy group: Rats were administered an IP injection of pilocarpine (40 mg/kg) and an ICV injection of saline, following the injection volumes mentioned above (this group was not included in the western blot analysis); (3) early Treatment group: Rats received an ICV microinjection of C34 (1 µg/rat) one hour after the IP injection of pilocarpine (40 mg/kg); (4)late treatment group: Rats were given an ICV microinjection of C34 (1 µg/rat) 24 hours after the IP injection of pilocarpine (40 mg/kg). All rats were sacrificed 48 hours after the pilocarpine/vehicle IP injection, and tissue preparations were conducted for histological and molecular analyses (Figure 1). 

### 3.3. Lithium-Pilocarpine Rat Model of Status Epilepticus

Rats received lithium chloride (LiCl) (127 mg/kg; Sigma-Aldrich) 20 hours prior to, and methylscopolamine (2 mg/kg, intraperitoneally, Sigma-Aldrich) 20 minutes before, the intraperitoneal injection of pilocarpine (40 mg/kg; Sigma-Aldrich). The rats' behaviors were monitored for approximately 2 hours to evaluate seizure stages. The Racine Scale was used to determine seizure stages: Stage 1, mouth and facial clonus; stage 2, head nodding; stage 3, forelimb clonus; stage 4, rearing; and stage 5, rearing and falling (13). Only animals that exhibited stage 5 generalized tonic-clonic seizures were selected as the status epilepticus (SE) model. To increase the survival rate and terminate seizures, diazepam (10 mg/kg, intraperitoneally) was administered 1 hour after SE induction.

### 3.4. Stereotaxic Surgery

**Figure 2.**
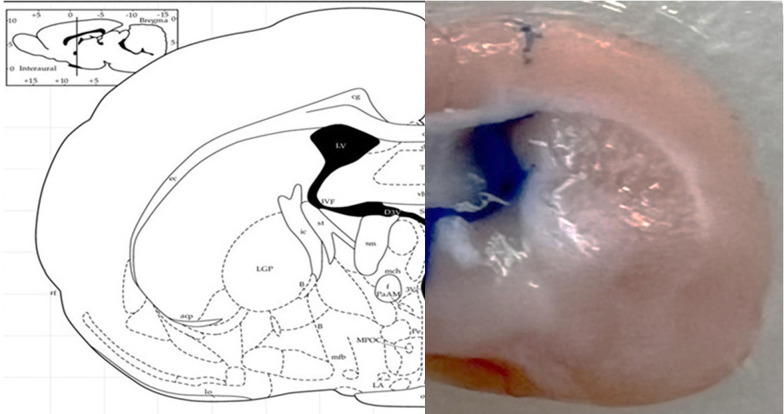
Representative image of brain section showing injection site and diffusion in the lateral ventricle.

The rats were anesthetized with an intraperitoneal injection of a mixture of ketamine 10% (100 mg/kg) and xylazine 2% (5 mg/kg). The rats were then secured in a stereotaxic frame, and a stainless steel guide cannula (23 gauge) was implanted above the right lateral ventricle at the following coordinates: Anterior-posterior (AP) -0.8, medial-lateral (ML) ± 1.5, dorsal-ventral (DV) -3.5, according to the Paxinos brain atlas. Stainless steel screws and acrylic cement were used to anchor the cannula to the skull. For the C34 microinjection, a 10 µL Hamilton syringe was used, connected to a gauge 30 needle via a short polyethylene tube. The injecting needle was 0.5 mm longer than the guide cannula. Methylene blue was injected to confirm the accuracy of cannula implantation and ICV injection (Figure 2). 

### 3.5. Hematoxylin and Eosin Staining

Rats were euthanized by CO2 asphyxiation and transcardially perfused with phosphate-buffered saline (PBS), followed by 4% paraformaldehyde (PFA) (Merck, Germany). The brains were carefully dissected, processed, and embedded in paraffin. Coronal sections with a thickness of 8 µm were prepared. The sections were deparaffinized and rehydrated in a descending series of alcohol concentrations. After treatment with xylene and graded alcohol, the samples were stained with hematoxylin for 10 minutes. Subsequently, the samples were incubated with eosin staining solution for 5 minutes. The samples were then dehydrated through graded alcohol. Finally, the washed sections were cover-slipped using a 90% glycerol mounting buffer. The hematoxylin and eosin (H&E)-stained sections were observed under a light microscope (Nikon, Japan).

### 3.6. Western Blotting

For western blotting analysis, the rats were euthanized using CO2 asphyxiation, and their brains were harvested. The hippocampi were rapidly dissected on ice and transferred to liquid nitrogen for 24 hours, then stored at -80^◦^C until molecular analysis. Initially, hippocampal tissues were homogenized in a lysis buffer containing a protease inhibitor cocktail (Roche, Germany). Protein concentration was measured using the Bradford assay. Pooled samples were prepared by mixing equal amounts of protein (µg of total protein) from each extracted sample. Twenty µg of total protein were electrophoresed in a 12% SDS-PAGE gel, transferred to a polyvinylidene difluoride (PVDF) membrane (Amersham Bioscience, UK), and probed overnight at 4^◦^C with monoclonal antibodies against TNF-α (1:1000 dilution; Santa Cruz Biotechnology, USA), caspase-3 (1:1000 dilution; Santa Cruz Biotechnology, USA), NF-κB (1:1000 dilution; Santa Cruz Biotechnology, USA), and β-actin (1:2000 dilution; Invitrogen, USA) as an internal control. After washing, the membrane was incubated with peroxidase-conjugated IgG (1:5000; Santa Cruz Biotechnology, USA) for 3 hours at room temperature. Immunoreactive polypeptides were detected by chemiluminescence using electrochemiluminescence reagents (Amersham Bioscience, UK) and subsequent autoradiography. Results were quantified by densitometry scanning of the films using ImageJ software, version 1.8. The relative level of the assessed polypeptide was expressed as the ratio of the polypeptide blot density to the β-actin blot density.

### 3.7. Statistical Analysis

Statistical analyses were conducted using GraphPad Prism 8 (GraphPad Software, CA, USA). The Shapiro–Wilk test was employed to assess the normal distribution of data. One-way ANOVA with the Tukey-Kramer multiple comparisons test was used to analyze the expression levels of TNF-α, caspase-3, and NF-κB. Results were expressed as mean ± standard error of the mean (SEM), and differences with P-values ≤ 0.05 were considered statistically significant.

## 4. Results

### 4.1. Early Treatment with C34, Toll-Like Receptor 4 Antagonist, Prevented Neural Death in Hippocampal Regions

In this study, the pilocarpine group exhibited a mortality rate of 52%. The late-treatment group had a mortality rate of 45%; however, the early-treatment group demonstrated a reduced mortality rate of 30%.

**Figure 3.**
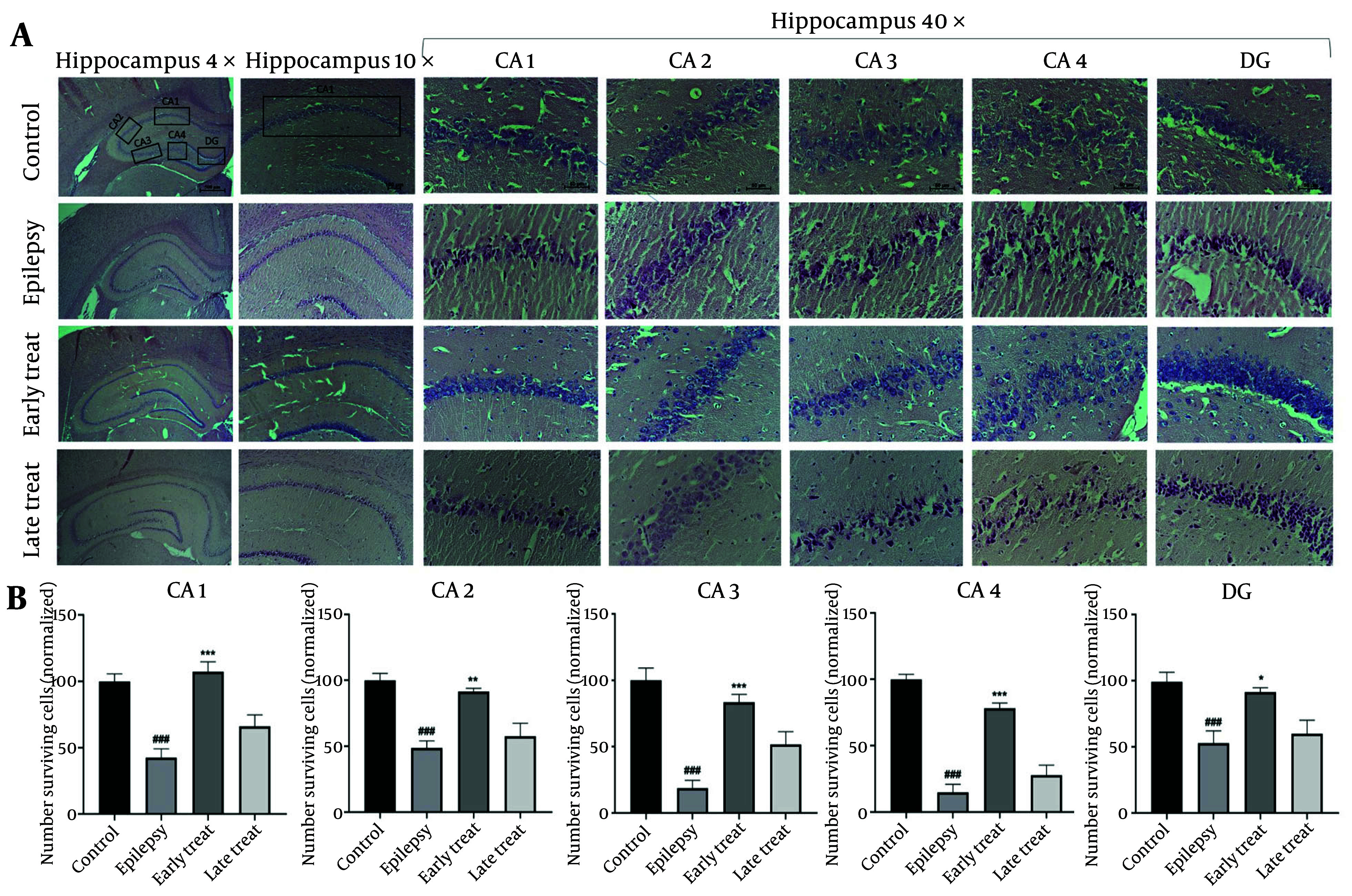
Hematoxylin and eosin (H&E) staining in rat hippocampus. A, the morphological changes of the hippocampal CA1/CA2/CA3/CA4/DG region post SE. B, the number of surviving neurons in the hippocampal CA1/CA2/CA3/CA4/DG region. Values shown are means ± SEMs for 3 rats (3 sections per rat). ### P < 0.001 (vs. control group); * P < 0.05, ** P < 0.01, *** P < 0.001 (vs. epilepsy group)

As shown in Figure 3A - C, the epilepsy group exhibited substantial histopathological changes, including neuronal atrophy and pyknosis across all hippocampal regions (CA1, CA2, CA3, CA4, and DG) compared to the control group (P < 0.001). The number of necrotic cells with pyknotic eosinophilic nuclei was significantly lower in the early-treatment group compared to the epilepsy group (P < 0.01). However, the late-treatment group did not show any significant improvement in hippocampal tissue morphology compared to the epilepsy group.

### 4.2. Early Administration of C34, a Toll-Like Receptor 4 Antagonist, Significantly Reduces Caspase-3, TNF-α, and NF-κB Levels, While Delayed Treatment Fails to Produce the Same Effect

**Figure 4.**
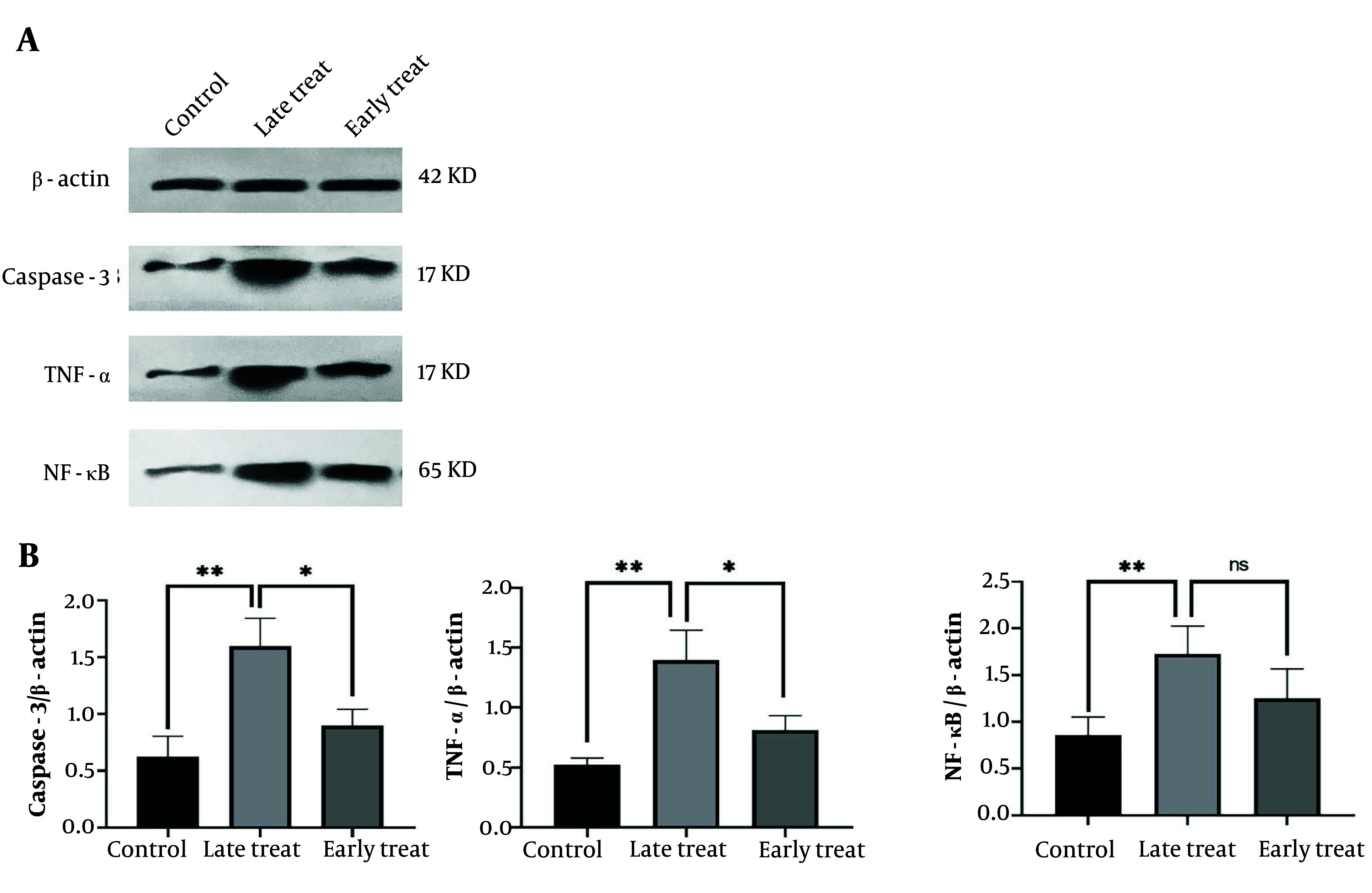
A, the representative western blots of ß-actin (internal control), caspase-3, TNF-α and ΝF-κΒ in the hippocampus; B, the mean ratio of each of caspase-3, TNF-α, and NF-κB to β-actin, respectively. Values shown are mean ± SEM of 3 repeats each representing the mean value of 6 rats per group, which analyzed by one-way ANOVA with Tukey multiple comparisons test. * P < 0.05, ** P ≤ 0.01; not significant).

As shown in Figure 4, the levels of caspase-3 and TNF-α significantly increased in the pilocarpine-injected group that received C34 24 hours after the SE model (late treatment group) (P ≤ 0.01) compared to the control group. However, in the group that received C34 one hour after the pilocarpine injection (early treatment group), the expression of caspase-3, TNF-α, and NF-κB significantly decreased compared to the late treatment group (P ≤ 0.05). Although the expression levels of caspase-3, TNF-α, and NF-κB in the early treatment group were slightly higher than those in the control group, this difference was not statistically significant.

## 5. Discussion

In our study, we investigated the neuroprotective effect of inhibiting TLR4 with C34 on hippocampal neuronal death and inflammatory factors during the acute period following SE. We compared early treatment with C34, administered one hour after pilocarpine injection, to late treatment, given 24 hours after the injection. We demonstrated that early treatment with C34 reduced the mortality rate and neuronal death induced by pilocarpine in the hippocampus. Additionally, it decreased the expression of caspase-3 and the neuroinflammatory factors TNF-α and NF-κB compared to the late treatment. These findings underscore the significance of early intervention in reducing neuronal death and decreasing inflammation in an epilepsy model.

In the current study, the epilepsy model was induced using pilocarpine intraperitoneal injection. Although this model is associated with high mortality rates, it remains one of the oldest and most well-studied animal models of SE (14). Epileptogenesis is thought to occur in three distinct stages: (1) The initial insult or acute period, (2) the latent period, and (3) the chronic epilepsy phase (15). In this study, during the acute period after SE, cell damage was observed in all areas of the hippocampus in rats that received pilocarpine. A recent study using Fluoro-Jade C staining showed that neurodegeneration in the hippocampal region, particularly prominent in the dentate gyrus, was evident 24 hours after SE and remained at the same level seven days later (16). This finding implies the critical importance of the first 24 hours following SE.

In a study by Fujikawa, neuronal damage resulting from SE induced by pilocarpine in rats was found to be progressive and varied across different brain regions. Initial signs of neuronal damage induced by pilocarpine appeared as minor damage in hippocampal regions after just 20 minutes of SE. This damage continued to evolve even after the seizures ceased, with the most severe damage observed 24 hours after SE, and cell breakdown observed after 72 hours (17). In addition to the progressive neuronal damage observed following pilocarpine-induced SE, another study demonstrated that severe and repetitive seizures during SE cause significant brain damage, particularly in vulnerable regions such as the hippocampus. This cumulative damage not only indicates the critical need for early intervention but also highlights its potential role in the subsequent development of chronic epilepsy (18). Therefore, the significantly reduced neuronal damage we observed in the hippocampus with early treatment (1 hour after SE) compared to late treatment (24 hours after SE) may be attributed to intervention before the progression of SE-induced damage.

Among the mechanisms underlying this progressive neural damage, neuroinflammation plays a crucial role. Early inflammatory processes in the brain have been reported to be associated with epilepsy (19). There is considerable evidence suggesting that neuroinflammation significantly contributes to seizure-induced neuronal death through various mechanisms, one of which involves the toll-like receptor (TLR) pathway (20, 21). We used C34 to inhibit TLR4 in the brain. C34, a 2-acetamidopyranoside with the formula C17H27NO9, has been shown through in silico docking studies to specifically inhibit TLR4 by directly binding to it. The inhibition of TLR4 signaling by C34 has been confirmed in both in vivo and in vitro studies (22). However, to the best of our knowledge, no study has investigated the intracerebral effects of C34 in an epilepsy model.

Toll-like receptor 4, when activated, stimulates nuclear factor kappa-B (NF-κB), which in turn activates inflammatory pathways (23). Our findings show that administering C34 one hour after the pilocarpine injection significantly reduced the expression of NF-κB and TNF-α in the hippocampus, compared to the treatment given 24 hours later. However, C34 administered 24 hours after pilocarpine injection could not decrease NF-κB or TNF-α expression as effectively as earlier treatment, indicating the critical role of neuronal inflammation in the pathophysiology of epilepsy, particularly during the very early stages following the initial insult.

Regarding the role of TLR4 and NF-κB in epilepsy, previous studies have demonstrated that the overexpression of HMGB1, which signals through TLR4 and activates NF-κB, exacerbates epileptogenesis (24). Similarly, Zhou et al. reported elevated levels of NF-κB expression in SE rats. They intracerebrally injected exogenous miR-322-5p, which binds to the 3′-UTR of TRAF6, a key component of the TLR4/NF-κB signaling pathway, and observed that the brain tissues of these SE rats exhibited reduced levels of inflammation and apoptosis in the hippocampal CA1 area (25). This is consistent with our results, where caspase-3, a crucial mediator of apoptosis, was decreased when TLR4 was inhibited by C34 one hour after the SE model compared to 24 hours after the SE model.

In a separate study, Wu et al. demonstrated that the expression of NF-κB in the hippocampus increased in a SE model of rats. Furthermore, administering a TLR4 inhibitor (TAK-242) intraperitoneally one hour after pilocarpine injection inhibited the activation of the inflammatory signaling pathway in microglia. They suggested that downregulating the TLR4/NF-κB inflammatory pathway may have antiepileptic effects in epilepsy (26). It has been shown that microinjection of exogenous damage-associated molecular patterns (DAMP) such as HMGB1 into the hippocampus in mice enhances the seizures induced by kainate through the stimulation of TLR4 (27). However, intravenous treatment with neutralizing anti-HMGB1 mAb inhibits inflammation in the very acute phase of SE induced by pilocarpine and reduces the apoptosis of pyramidal cells (28).

It has been demonstrated that TLR4 mediates microglial activation and induces NF-κB activation, playing a critical role in the activation of the M1 microglial phenotype. Inhibiting the pilocarpine-induced TLR4/NF-κB pathway in the hippocampi of epileptic rats promotes the polarization of microglia to the M2 phenotype, suggesting this pathway as a potential strategy for alleviating epileptogenesis (29). It can be assumed that C34 may have a therapeutic effect in the SE model of epilepsy by inhibiting the TLR4/NF-κB inflammatory pathway.

In the context of early treatment strategies, Rosciszewski et al. reported that blocking HMGB-1 with glycyrrhizin immediately after pilocarpine-induced SE in rats led to a reduction in neuronal degeneration, reactive astrogliosis, and microgliosis over the long term (30). Their results align with ours, as we demonstrated that inhibiting TLR4 one hour after pilocarpine-induced SE significantly reduced neurodegeneration in the hippocampus. In this study, we also observed that inhibiting TLR4 with the compound C34, when administered 24 hours after pilocarpine-induced SE, does not effectively prevent neurodegeneration in the hippocampus. This finding suggests that neuroinflammation occurring in the early minutes post-insult may activate molecular pathways that initiate and propagate fundamental and progressive changes in the brain. The early activation of these inflammatory pathways underscores the importance of timely therapeutic intervention to mitigate long-term neurodegenerative consequences and improve clinical outcomes in epilepsy.

In this line, Suleymanova et al. demonstrated a neuroprotective effect by antagonizing CB1 receptors, significantly reducing the number of neurodegenerating neurons four hours post-SE during the acute phase. However, it is noteworthy that this neuroprotective effect did not persist into the latent period (16). Our study highlighted the critical importance of precise timing within the early minutes of the acute phase, demonstrating that even slight variations at specific time points during this phase can significantly influence levels of neurodegeneration. Specifically, inhibiting TLR4 during the critical first minutes after SE resulted in a significant decrease in the number of neurodegenerating neurons across all areas of the hippocampus, compared to inhibiting TLR4 24 hours after SE in the acute period.

One of the limitations of this study is the absence of a standalone epilepsy (pilocarpine-only) group in the western blot analysis, which would have allowed for a more direct comparison of cytokine expression levels across all experimental conditions. While this group was included in the histological analysis, where it clearly demonstrated that the rate of neuronal death in different regions of the hippocampus was quite similar to that observed in the late C34 treatment group, its inclusion in the western blot analysis would have provided additional insights into the molecular changes among groups. Although our previous study demonstrated that pro-inflammatory cytokine levels were elevated in the rat hippocampus 24 hours after pilocarpine administration (31), including a pilocarpine-only group in the western blot analysis would have allowed for a more accurate comparison of cytokine expression between the early and late C34 treatment groups and untreated epileptic animals. This would have helped to further elucidate the extent to which C34 modulates inflammatory pathways at different treatment time points. However, due to experimental constraints, this analysis was not conducted. We acknowledge this as a limitation, emphasizing the need for future studies to incorporate a dedicated epilepsy control group in molecular analyses for a more comprehensive understanding of treatment effects.

### 5.1. Conclusions

Our results indicate the critical role of neuronal inflammation in the pathophysiology of epilepsy, emphasizing how inflammatory processes within neurons are pivotal during the very early stages following the initial insult. This early intervention window is crucial for mitigating the inflammatory cascade that contributes to the progression and severity of epileptic episodes. By addressing neuronal inflammation promptly, we can gain a better understanding of its impact on the development of epilepsy and potentially improve therapeutic strategies to manage and prevent epileptic conditions more effectively.

## Data Availability

The dataset presented in the study is available on request from the corresponding author during submission or after publication.
